# Integrating Life Stages into Ecological Niche Models: A Case Study on Tiger Beetles

**DOI:** 10.1371/journal.pone.0070038

**Published:** 2013-07-23

**Authors:** Angela Taboada, Henrik von Wehrden, Thorsten Assmann

**Affiliations:** 1 Institute of Ecology, Leuphana University Lüneburg, Lüneburg, Germany; 2 Area of Ecology, Department of Biodiversity and Environmental Management, University of León, León, Spain; 3 Centre of Methods, Leuphana University Lüneburg, Lüneburg, Germany; 4 Research Institute of Wildlife Ecology, Vienna, Austria; USDA-Agricultural Research Service, United States of America

## Abstract

Detailed understanding of a species’ natural history and environmental needs across spatial scales is a primary requisite for effective conservation planning, particularly for species with complex life cycles in which different life stages occupy different niches and respond to the environment at different scales. However, niche models applied to conservation often neglect early life stages and are mostly performed at broad spatial scales. Using the endangered heath tiger beetle (*Cicindela sylvatica*) as a model species, we relate presence/absence and abundance data of locally dispersing adults and sedentary larvae to abiotic and biotic variables measured in a multiscale approach within the geographic extent relevant to active conservation management. At the scale of hundreds of meters, fine-grained abiotic conditions (i.e., vegetation structure) are fundamental determinants of the occurrence of both life stages, whereas the effect of biotic factors is mostly contained in the abiotic signature. The combination of dense heath vegetation and bare ground areas is thus the first requirement for the species’ preservation, provided that accessibility to the suitable habitat is ensured. At a smaller scale (centimetres), the influence of abiotic factors on larval occurrence becomes negligible, suggesting the existence of important additional variables acting within larval proximity. Sustained significant correlations between neighbouring larvae in the models provide an indication of the potential impact of neighbourhood crowding on the larval niche within a few centimetres. Since the species spends the majority of its life cycle in the larval stage, it is essential to consider the hierarchical abiotic and biotic processes affecting the larvae when designing practical conservation guidelines for the species. This underlines the necessity for a more critical evaluation of the consequences of disregarding niche variation between life stages when estimating niches and addressing effective conservation measures for species with complex life cycles.

## Introduction

Ecological niche modelling (ENM) tools are increasingly applied to address a miscellany of challenges in species conservation biology and distributional ecology (e.g., estimation of extinction risks, planning of reserve networks, prediction of climate change effects) [1]. When applying ENMs to provide scientific guidance and effectively inform conservation practitioners, it is essential, inter alia, to fully understand: 1) which are the fundamental factors defining a species’ niche and geographic distribution [1], [2], 2) what is the relative importance of such factors across spatial scales (i.e., at multiple extents and resolutions) [1], and 3) how much variation in life strategy and ecological niche exists over the complete life cycle of a species [3]–[5].

In this emerging field, the theoretical framework by Peterson *et al.* [1] and Soberón [2] brings conceptual clarity into the relationship between niches and distributional areas (see also [6]). In practice, the multidimensional view of the niche concept [7] is split into two operational niche classes [1], [6], [8], [9]: 1) the Grinnellian niche [10] defined by scenopoetic variables (abiotic factors) not affected by the species and acting at large (e.g., continental or regional) scales; and 2) the Eltonian niche [11] defined by bionomic variables (biotic interactions and resources) dynamically linked to the population levels of the species and acting at small (e.g., local or site) scales. However, evidence that biotic factors display significant spatial structure and correlate closely with abiotic factors [12] challenges the validity of this simplified partitioning of the two types of variables and poses an unsolved question in ENM (see [6]). Further empirical confirmation is, therefore, required [2] to support this hierarchical view of unlinked abiotic and biotic factors shaping species distributions across spatial resolutions (e.g., [9]). Indeed, abiotic factors may still be crucial at very small scales for certain organisms (e.g., ants [13]; moths and flies [14]; butterflies [15]; ground beetles [16]), whereas there are examples which corroborate either negligible [17] or significant [5], [18]–[21] and species-dependent [22] effects of biotic processes at large scales.

Basically, the spatial extent and resolution of ENM should match the extent and resolution of the biological phenomenon under study and also take into account the species’ natural history and dispersal [1], [6], [23]. To date, however, most ENM studies have been developed at global to regional scales [1], owing to the number of widely available databases recording species occurrences (e.g., natural history collections) and abiotic conditions (e.g., climatic data) over large areas and at coarse resolutions [24]. For many species, inclusion of accurate biotic information in such broad-scale models is unfeasible [6], since the biotic niche component is too fine-grained and dynamic (e.g., [12]) to be mapped at high resolution over the entire distribution range of a species. Such a shortcoming may be critical for organisms with complex life histories (e.g., holometabolous insects; see [25]), for which detailed biological information is essential to avoid misleading interpretations of ENM results [3], [26], [27]; this is particularly important when considering conservation actions [4].

Whether models for species with complex life histories should take more than a single life stage into account is a pending issue in ENM, as the persistence of viable populations is only feasible when the abiotic and biotic requirements of every life stage are fulfilled [3], [4]. In general, compared to adults, early life stages have more specialised needs and less mobility (e.g., ground beetles [28]; butterflies [3]), are more vulnerable to environmental changes ([27]; see also [29]), and very likely display different biotic interactions [30]–[32]. Rewording the rationale by Pearman *et al.* [33], there seems little doubt that achieving detailed understanding of niche variation among life stages is a key aspect in ENM: 1) models that are developed by pooling across life stages within species or by omitting early-stage constraints may ignore the possibility that each life stage occupies a distinct niche, and thus disregard the potentially different responses of life stages to the environment [3], [34]; 2) a species model could overvalue the potential niche and species-level response to changes in the environment when, in fact, it is impossible for all life stages to cope with such changes [27], [29], [35]; and 3) the portion of a species range (and suitable habitat) that is occupied by the different life stages can vary greatly [25], [30] or be roughly coincidental [36], depending on the specific needs, strength of interactions and dispersal restrictions of each life stage.

In this study we identify the factors that define life-stage ecological niches at fine resolution, by taking into account the life-history traits (e.g., body size and mobility), and robust presence/absence and abundance data of co-occurring larvae and adults. We use tiger beetles as model organisms in a conservation approach to address the following questions: 1) Do co-occurring life stages exhibit identical fine-grained responses to the same environmental variables? 2) Are biotic factors (i.e., intra- and interspecific interactions) more relevant than abiotic ones in shaping life-stage distribution at fine spatial resolution? 3) Does the explicit inclusion of life-stage information affect the interpretation of species niches and distributional areas?

## Materials and Methods

### Ethics Statement

Permits and approvals for the field campaign were obtained from the Regional Administration (Junta de Castilla y León EP/LE/376/2011, and Gobierno del Principado de Asturias 2011/009739–2012/001887), and in consultation with all shepherds of the study sites.

### Study Species

The heath tiger beetle, *Cicindela sylvatica* Linnaeus, 1758 (Coleoptera: Carabidae), is an endangered stenotopic species occurring in north, central and north-west Europe [37], with isolated populations in northern Spain [38]. These populations inhabit rather small and fragmented habitat patches in subalpine areas or isolated montane valleys.

The species’ entire life cycle (i.e., from egg to adult) takes place in the same location. Larvae go through three instars before pupation [39] and larval development is most likely completed within a 1–3 year period (see [40]). First, second and third instar larvae co-occur due to differences in developmental speed between individuals (see [41]). Adults (15–19 mm length) may live for one or two further years. Both larvae and adults are diurnal opportunistic predators, feeding on surface-active invertebrates (e.g., ants, lepidopteran larvae, wasps) [37], and primarily thriving in sun-exposed bare areas. Sedentary larvae live in vertical burrows built in the oviposition substrate chosen by the female. Larvae are generally active in May-September, but, during adverse weather, they close the burrows and become inactive. Only in extreme conditions (e.g., desiccation, flooding) may the larvae leave the burrows and relocate [39]. Adult beetles are rapid runners and agile fliers, and are mainly active in May-July. In spite of the adults’ dispersal abilities, long distance movements have not been reported in the literature and the maximum linear distance recorded, travelled by a single individual, is ca. 200 m [42]. However, it is possible that adults move at least 500 m [43] or even up to 700 m [42] across suitable habitat.

In the study area, the target species co-occurs with the green tiger beetle, *Cicindela campestris* Linnaeus, 1758, a eurytopic species, widely distributed across the entire Palaearctic region [37]. This species exhibits similar biology [39], seasonal activity and dispersal abilities (see [42]) to the target species.

### Study Area

**Figure 1:**
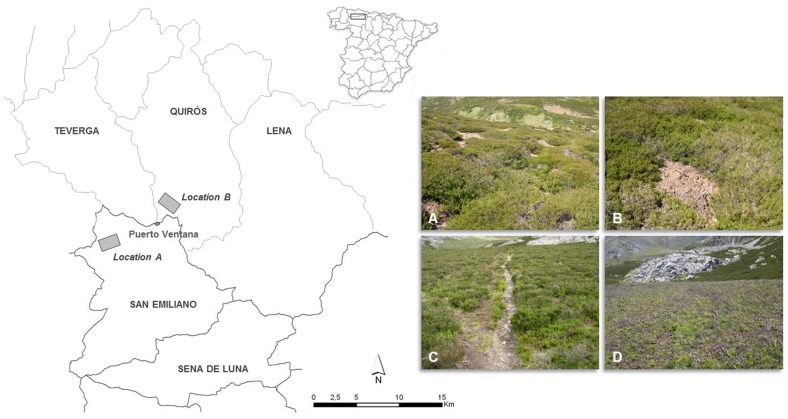
Study locations (A and B) in the Cantabrian mountain range, NW Spain (43 °**1**′**–43**°**4**′**N, 5**°**59**′**–6**°**6**′**W).** Photographs depict the four *Calluna vulgaris* heathland habitat types defined by different structures of the vegetation and bare ground mosaic. A = BARE_CUSHION, B = BARE_SPOT, C = BARE_PATH and D = BURNED. See text for further explanation.

We surveyed two locations (A: 43°1′27″N–6°6′34″W, 1860 m a.s.l., 9 ha; and B: 43°4′29″N–5°59′18″W, 1775 m, 15 ha) in the Cantabrian mountain range (NW Spain), at the rear edge of the species’ distribution range (Figure 1). For centuries, the area was grazed in summer by local and transhumant sheep flocks, at present, however, it is used for raising cattle (ca. 50 cows per location).

### Sampling Methods

We used a proportional random-stratified approach (see [24]) to gather field data. We identified the main habitat types that are believed to represent meaningful environmental gradients for the species (Figure 1; see [1], [24]). Habitat types in location A are: 1) BARE_CUSHION: *Calluna vulgaris* (L.) Hull heathland characterised by the combination of dense vegetation arranged in a cushion-like structure and large patches of bare ground, 2) BARE_SPOT: *C. vulgaris* heathland dominated by dense vegetation interrupted by small spots of bare ground, 3) DENSE: *C. vulgaris* heathland consisting of dense continuous vegetation, 4) GRASSLAND: upland siliceous grassland used as summer pasture, and 5) SHRUBLAND: shrub formation consisting of *Cytisus purgans* (L.) Boiss, *Cytisus scoparius* (L.) Link and *Genista florida* L. Habitat types in location B are: 1) BARE_PATH: *C. vulgaris* heathland consisting of dense vegetation interrupted by bare ground paths caused by cattle trampling, 2) BURNED: *C. vulgaris* heathland burned in September 2010 to create pastureland and improve forage quality, 3) DENSE, 4) GRASSLAND and 5) SHRUBLAND.

We randomly placed a total of 120 sampling points in each location. The number of sampling points per habitat type was proportional to the expected relevance of each habitat for the species according to the literature and to the spatial extent of the habitat (Table S1). In each habitat, sampling points were spaced a minimum distance of 5.5 m apart to assure independent observations (see [44]).

**Figure 2:**
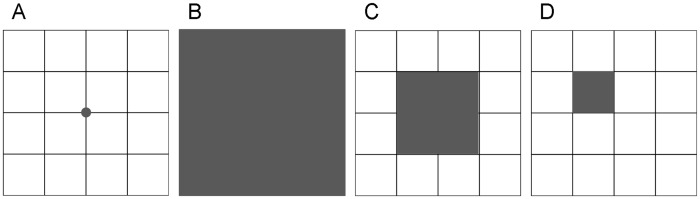
Schematic representation of the sampling units used to survey adults and larvae at each sampling point. Adults were captured by pitfall trapping (i.e., one pitfall trap per sampling point; panel A) and larvae were mapped at three spatial scales: 1×1 m quadrat (represented by the shaded area in panel B), 50×50 cm quadrat (represented by the shaded area in panel C) and 25×25 cm quadrat (the shaded area in panel D exemplifies one of the 16 quadrats mapped at each sampling point).

Adult beetles were captured by live pitfall trapping in July 2011. We placed one dry trap consisting of a large plastic cup (85 mm diameter, 100 mm high) containing an inverted medium-sized plastic cup (62 mm diameter, 88 mm high) per sampling point (Figure 2) (see [45] for details). We emptied the traps on a daily basis, alternately in the two locations (i.e., 11 and 14 capture events, and 22 and 28 total trapping days in locations A and B, respectively).Trapped adults were marked individually with unique number-coded combinations of dots in the elytra using a small drill, and released.

Beetle larval stages were surveyed at three spatial scales (i.e., 1×1 m, 50×50 and 25×25 cm quadrats; Figure 2, Table 1) at each sampling point. The multiscale approach used to survey larvae was aimed at describing the appropriate resolution at which larvae are influenced by the environment and at detecting the extent of crowding effects (i.e., the scale at which neighbouring larvae may influence each other). We mapped the exact burrow positions in the quadrats and monitored the larvae twice (June and July 2011) to identify occasional burrow closure and obtain a single complete map of larval occurrence for each sampling point. Co-occurring first, second and third larval instars (hereafter, larva_1, larva_2 and larva_3) were determined by measuring the diameter of the burrow opening, which correlates to the size of the head and prothorax of the larval instars ([40]; see also [41]). We assessed larval neighbourhood influence by estimating the number of larvae inhabiting the adjacent 25×25 cm quadrats (i.e., 12 quadrats bordering the sampled 50×50 cm quadrat, and between 3 and 8 quadrats bordering the sampled 25×25 cm quadrats; see Figure 2 and Table 1). The larvae of the two tiger beetle species were distinguished by careful observation of the head and prothorax (see [46]) while they were settled at the surface opening of the burrows or after extracting them by the fishing technique [47].

**Table 1:** Data sets resulting from the combination of life stage and sampling scale.

Data set	Life stage	Response	*N*	Sampling unit	Abiotic predictors (scale)	Biotic predictors (sampling unit)
1 - 2	Adult; Male; Female	PA, AB; PA;PA	120	Pitfall trap	COVER (50×50 cm, 1×1 m quadrat); RESISTANCE (1×1 m quadrat); SOIL (1×1 m quadrat); STRUCTURE(3 m, 6 m radius)	Larva PA, AB (1×1 m quadrat); Larva_1 AB (1×1 m quadrat ); Larva_2 AB (1×1 m quadrat ); Larva_3 AB (1×1 m quadrat ); Congeneric adult PA, AB (trap)
3 - 4	Larva; Larva_1; Larva_2; Larva_3	PA, AB; AB;AB; AB	120	1×1 m quadrat	COVER (1×1 m quadrat); RESISTANCE (1×1 m quadrat); SOIL (1×1 m quadrat); STRUCTURE (3 m, 6 m radius)	Adult PA, AB (trap); Male PA (trap); Female PA (trap); Congeneric larva PA, AB (1×1 m quadrat)
5 - 6	Larva; Larva_1; Larva_2; Larva_3	PA, AB; AB;AB; AB	120	50×50 cm quadrat	COVER (50×50 cm, 1×1 m quadrat); RESISTANCE (1×1 m quadrat); SOIL (1×1 m quadrat); STRUCTURE(3 m, 6 m radius)	Adult PA, AB (trap); Male PA (trap); Female PA (trap); Neighbouring larva AB (bordering 25×25 cm quadrats)
7 - 8	Larva	PA, AB	160, 176	25×25 cm quadrat	COVER (25×25 cm, 1×1 m quadrat); RESISTANCE (1×1 m quadrat); SOIL (1×1 m quadrat); STRUCTURE(3 m, 6 m radius)	Adult AB (trap); Neighbouring larva AB (bordering 25×25 cm quadrats)

Target species = *Cicindela sylvatica*, congeneric species = *Cicindela campestris*. Data sets 1, 3, 5 and 7 correspond to location A, and 2, 4, 6 and 8 to B. Adults were captured by pitfall trapping and larvae were surveyed at three spatial scales (i.e., 1×1 m, 50×50 and 25×25 cm quadrats) (see Figure 2). The response variables (PA = presence/absence, AB = abundance) and the abiotic and biotic predictor variables included in the models are indicated for each data set. *N* = number of observations. Larva_1, 2, 3 = first, second, and third larval instars, respectively. COVER = percentage cover, RESISTANCE = vegetation resistance, SOIL = soil features, STRUCTURE = habitat structure; see Table 2.

The combination of sampling location, life stage and scale yielded the following data sets (Table 1): 1) location A, adult; 2) B, adult; 3) A, larva, 1×1 m quadrat; 4) B, larva, 1×1 m; 5) A, larva, 50×50 cm; 6) B, larva, 50×50 cm; 7) A, larva, 25×25 cm; and 8) B, larva, 25×25 cm. All data sets included 120 observations, except the smallest scale (data sets 7 and 8). For these data sets, we included only the sampling points with a minimum number of mapped larvae during the first larval survey (i.e., 5 and 3 larvae per sampling point or 16 25×25 cm quadrats in locations A and B, respectively), in order to account for the potential influence of neighbourhood density on larval occurrence, which is expected to arise at the scale of few centimetres [41]. In total, ten and eleven sampling points (i.e., 160 and 176 observations or 25×25 cm quadrats) with the highest number of larvae were chosen among the 120 sampling points in locations A and B, respectively.

Environmental information (Table 2) was gathered at different spatial scales corresponding to the resolution of the adult and larval surveys and to their dispersal abilities. We determined 32 variables relevant to the species, and grouped these into four categories: percentage cover (COVER), vegetation resistance (RESISTANCE), soil features (SOIL) and habitat structure (STRUCTURE).

**Table 2:** Abiotic predictor variables grouped in four categories (COVER, RESISTANCE, SOIL and STRUCTURE) and measured at different spatial scales.

Category	Scale	Abbreviation	Description	Unit
Percentage cover (COVER)	25×25 cm quadrat, 50×50 cm quadrat, 1×1 m quadrat	BARESOIL_25, BARESOIL_50, BARESOIL_1	Bare soil cover	%
		CALLUNA_25, CALLUNA_50, CALLUNA_1	*Calluna vulgaris* cover	%
		DUNG_25, DUNG_50, DUNG_1	Cattle dung cover	%
		GRAMINOID_25, GRAMINOID_50, GRAMINOID_1	Graminoid species cover	%
		HERB_25, HERB_50, HERB_1	Total herb species (including graminoids) cover	%
		LICHEN_25, LICHEN_50, LICHEN_1	Lichen cover	%
		LITTER_25, LITTER_50, LITTER_1	Leaf litter cover	%
		MOSS_25, MOSS_50, MOSS_1	Moss cover	%
		ROOT_25, ROOT_50, ROOT_1	Root (alive and dead) cover	%
		SEDUM_25, SEDUM_50, SEDUM_1	*Sedum album* cover	%
		SHRUB_25, SHRUB_50, SHRUB_1	Total shrub species (including *Calluna vulgaris*, V*accinium myrtillus* and *V. uliginosum*) cover	%
		STONE_25, STONE_50, STONE_1	Stone cover	%
		VACCINIUM_25, VACCINIUM_50, VACCINIUM_1	*Vaccinium myrtillus* and *V. uliginosum* cover	%
		WOOD_25, WOOD_50, WOOD_1	Dead wood cover	%
Vegetation resistance (RESISTANCE)	1×1 m quadrat	COVER_N, COVER_E, COVER_S, COVER_W	Vegetation cover estimated in a 6×6 square grid (0.13 m^2^) vertically centered in the quadrat at each cardinal direction	%
		HEIGHT	Mean (*N* = 7) *Calluna vulgaris* height	cm
		SQUARE_N, SQUARE_E, SQUARE_ S, SQUARE_W	Number of squares crossed by vegetation in a 6×6 square grid (0.13 m^2^) vertically centered in the quadrat at each cardinal direction	
Soil features (SOIL)	1×1 m quadrat	LITTER_DEPTH	Mean (*N* = 5) depth of the leaf litter layer	cm
		SOIL_0.063	Soil (horizon A) particle size <0.063 mm	%
		SOIL_0.125	Soil (horizon A) particle size 0.063–0.125 mm	%
		SOIL_0.25	Soil (horizon A) particle size 0.125–0.25 mm	%
		SOIL_0.50	Soil (horizon A) particle size 0.25–0.50 mm	%
		SOIL_1	Soil (horizon A) particle size 0.50–1 mm	%
		SOIL_2	Soil (horizon A) particle size 1–2 mm	%
		SOIL_HUMID	Soil (horizon A) humidity	%
		SOIL_OM	Soil (horizon A) organic matter content	%
		SOIL_PH	Soil (horizon A) pH	
Habitat structure (STRUCTURE)	3 m radius, 6 m radius	OPEN_3, OPEN_6	Number of bare ground patches	
		SHRUB_3, SHRUB_6	Number of shrubs >1 m height	
		STRUCTURE_3, STRUCTURE_6	Habitat structure classes: 1) 100% closed vegetation (*Calluna vulgaris*), 2) few distant open bare ground patches, 3) 50% open - 50% closed patches, 4) mostly open patches, 5) 100% open patches, 6) 100% closed vegetation (shrub species >1 m height)	
	6 m radius	DIST_OPEN	Distance to the nearest bare ground patch	cm
		DIST_SHRUB	Distance to the nearest shrub >1 m height	cm

### Response Variables

We modelled the ecological niche of the species, separately for each life stage (adult and larva), and also, the partial niches of sexes (male and female) and larval instars (larva_1, larva_2 and larva_3) (see [26], [28]). We analysed the following response variables: 1) presence-absence data (PA), where adult presence was recorded by the traps and larval presence on the maps, and 2) abundance data (AB), as the sum of distinctively identified individuals (i.e., individually marked adults and monitored larvae) recorded by the traps and on the maps. We modelled PA responses for adult, male, female and larva, and AB responses for adult, larva, larva_1, larva_2 and larva_3 (Table 1).

### Abiotic Predictor Variables

For each data set, an identical group of environmental variables was evaluated as abiotic predictors of the occurrence of the target species (Table 1). Multiple scales of measurement of the COVER and STRUCTURE environmental variables (Table 2) were considered simultaneously in the models (Table 1). The combination of these different scales is expected to improve model performance [13], [18], [36]. After selection of variables (see below), only the most relevant scale for each predictor was retained in the models.

### Biotic Predictor Variables

Assuming unlimited prey resources, we evaluated the main potential intra- and interspecific interactions (either positive or negative) between: 1) adults and larvae of the target species, 2) adults of the target and congeneric species (hereafter, congeneric adults), 3) larvae of the target and congeneric species (hereafter, congeneric larvae), and 4) larvae and neighbouring larvae of the target species. The following variables were included as biotic predictors in the models (Table 1): larva (PA, AB) and congeneric adult (PA, AB) in the adult models; adult (PA, AB) and congeneric larva (PA, AB) in the larval models at 1×1 m; adult (PA, AB) and neighbouring larva (AB) in the larval models at 50×50 and 25×25 cm.

### Data Analysis

We fitted generalised linear models (GLMs) to describe the ecological niche of the adult and the larva, and the partial niche of sexes and larval instars. Partial niche models yielded analogous outcomes and had lower qualities than the adult and larval models; they are thus not reported. We initially performed generalised linear mixed models (GLMMs) for the finest larval scale (25×25 cm), considering the identity of the 1×1 m square as random factor; however, the increase in model complexity did not improve predictive power and therefore only GLMs are reported.

We modelled PA data following a binomial error distribution (or quasibinomial distribution to account for overdispersion), using the logit link function; and AB data following a negative binomial error distribution (or Poisson distribution in the case of high values of the clumping parameter *k*, i.e., low degree of aggregation), using the log link function [48]. For each response variable, we calibrated three GLMs using different sets of predictor variables (see [21]): 1) only the abiotic (ABIOT), 2) only the biotic (BIOT) and 3) the combination of abiotic and biotic predictors from the ABIOT and BIOT models (FULL).

For each data set, the same predictors were initially included in the ABIOT and BIOT models (Table 1). The starting sets of predictors consisted of variables that resulted in univariate GLMs with *p*<0.1 and with the highest explained deviance. To avoid collinearity, only uncorrelated variables (Spearman’s rank correlation coefficient −0.7<*ρ*<0.7) were considered in the models. Additionally, variable selection was based on the expected relevance of the predictors for the target species accounted for in the literature.

Collinearity was further assessed in the models by examining the variance inflation factor (VIF) of the predictors [24], [49]. We sequentially dropped the covariate with the highest VIF from the models, until all predictor VIFs were smaller than the preselected threshold value of 10. Minimal adequate models (MAMs) to describe the data were determined by both backward and forward stepwise variable selection based on Akaike’s information criterion (AIC). We measured the goodness of fit of the ABIOT and BIOT MAMs to the data by Nagelkerke’s coefficient of determination (*R^2^*). For the ABIOT MAMs, we calculated the joint percentage of deviance explained by the predictors in each category (COVER, RESISTANCE, SOIL and STRUCTURE). We checked for spatial autocorrelation in the model residuals by calculating Moran’s index (*I*). We tested Moran’s *I* significance by performing 1000 permutations and applying Holm’s correction to adjust for repeated testing. We found weak evidence for spatial autocorrelation in the species distribution, as only two out of 32 performed models had significantly clumped residuals (see [16]).

Two approaches were applied to evaluate the accuracy of the predictions of the ABIOT models [24]: 1) internal evaluation (IE), using a single data set to calibrate and evaluate the model by running 2-fold (i.e., random 50% split) cross-validations (CVs) iterated 100 times, and 2) external evaluation (EE), using two independent calibration-evaluation sets of data from locations A and B (e.g., data set 1 was used for model fitting and data set 2 for model evaluation, and *vice versa*). We evaluated the accuracy of the predictions of the BIOT models by IE. For PA response variable models, predictions were compared to observations by applying the area under the receiver operating characteristic (ROC) curve (AUC). The AUC is an informative measure of model accuracy when estimating the realised niche of a species and when true absence data are available [50]. AUC values range from 0 to 1, with a model discriminating better than chance if AUC>0.50. Following Araújo *et al.* [51] and Randin *et al.* [52], AUC values were interpreted as: excellent AUC>0.90; good 0.80<AUC<0.90; fair 0.70<AUC<0.80; poor 0.60<AUC<0.70; fail 0.50<AUC<0.60. For models calibrated with AB response variables we compared predictions to observations by the Spearman’s rank correlation coefficient *ρ* [24]. Similarly, to help interpret the agreement between predictions and observations measured by *ρ*, the following ranges were established, taking into account the critical value of the correlation coefficient with *N*>100 [53]: excellent *ρ*>0.80; good 0.60<*ρ*<0.80; fair 0.40<*ρ*<0.60; poor 0.20<*ρ*<0.40; fail *ρ*<0.20.

We evaluated transferability (T) of the ABIOT MAMs between life stages (data sets 1–2 and 5–6) with the AUC and Spearman’s *ρ* measures for PA and AB data, respectively. Transferability was assumed to fail when AUC<0.70 and *ρ*<0.40. Adult model transferability (i.e., ability of adult models to predict larval occurrence) was evaluated by assessing
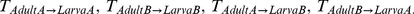
and larval model transferability (i.e., ability of larval models to predict adult occurrence) was evaluated by assessing



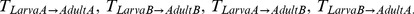



We checked for asymmetrical transferability (AT, see [52]): 1) between locations (AT_Location_) by calculating the difference in model T (mean AUC and *ρ* values) from location A to B and from B to A as
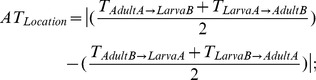
and 2) between life stages (AT_Stage_) by calculating the difference in model T from adult to larva and from larva to adult in the same location as
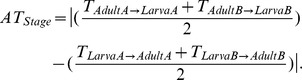



We compared ABIOT, BIOT and FULL models using the AIC and Nagelkerke’s *R^2^*. The model with the lowest AIC value and the maximal deviance reduction indicates the best fit to the data. Following previous work [21], [54], [55], we used a hierarchical partitioning approach to estimate the contribution of the abiotic and biotic predictor sets by subtracting the *R^2^* of the opposite set from the FULL model. The pure contribution of the abiotic and biotic predictors to the total explanatory power is defined by.
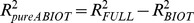
and



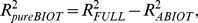
respectively. The joint contribution of the two predictor sets was calculated as



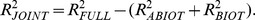



We interpret the niche model outcomes graphically, by integrating life-stage constraints into a simplified representation of the **BAM** diagram [8], [56], a heuristic tool that incorporates the three major elements which determine species distributions [2], [57]: biotic interactions (**B**), abiotic conditions (**A**), and movements (**M**; i.e., dispersal and colonisation factors; see [9]).

All data analyses were carried out with R software, version 2.14.0 [58] using the ‘MASS’ [59], ‘car’ [60], ‘fmsb’ [61], ‘ncf’ [62] and ‘verification’ [63] packages.

## Results

### Life-stage Occurrence

The total number of adult captures and monitored larvae was 118 and 383 in location A, and 82 and 174 in B, respectively (Table S1). Adults occurred in 35% of the traps, whereas larvae were recorded in 45% of the sampling points. In location A, both life stages occurred almost exclusively in the BARE_CUSHION habitat type. In location B, the majority of adults and all the larvae were found in the BARE_PATH habitat type.

### Abiotic Model

The fit of PA models was always lower than that of AB models (Table 3). In 13 of the 16 ABIOT MAMs, final predictors explained more than 50% of the null deviance. The measures of fit (*R^2^* values) of the adult models ranged from 0.62 to 0.91. The fit of the larval models was highest at 1×1 m (0.81–0.94) but values decreased with increasing spatial resolution (0.67–0.83 at 50×50 cm and 0.34–0.55 at 25×25 cm).

**Table 3:** Performance (Nagelkerke’s *R^2^*) of the abiotic (ABIOT), biotic (BIOT) and combined (FULL) models for each adult and larval presence/absence (PA) and abundance (AB) data set of the target species *Cicindela sylvatica*.

Data set	PA			AB		
	ABIOT	BIOT	FULL	ABIOT	BIOT	FULL
**Adult**						
1	0.73	0.53	**0.76**	0.91	0.73	**0.93**
2	0.62	0.20	**0.65**	0.78	0.20	**0.79**
**Larva**						
3	0.81	0.61	**0.85**	0.91	0.73	**0.94**
4	0.93	0.16	**0.94**	**0.94**	0.03	**0.94**
5	0.67	0.63	**0.76**	0.83	0.64	**0.87**
6	**0.67**	0.28	**0.67**	0.81	0.49	**0.82**
7	**0.40**	0.13	**0.40**	**0.55**	0.12	**0.55**
8	0.34	0.14	**0.35**	**0.43**	0.15	**0.43**

Bold face indicates the model with the highest fit (i.e., maximal deviance reduction).

**Figure 3:**
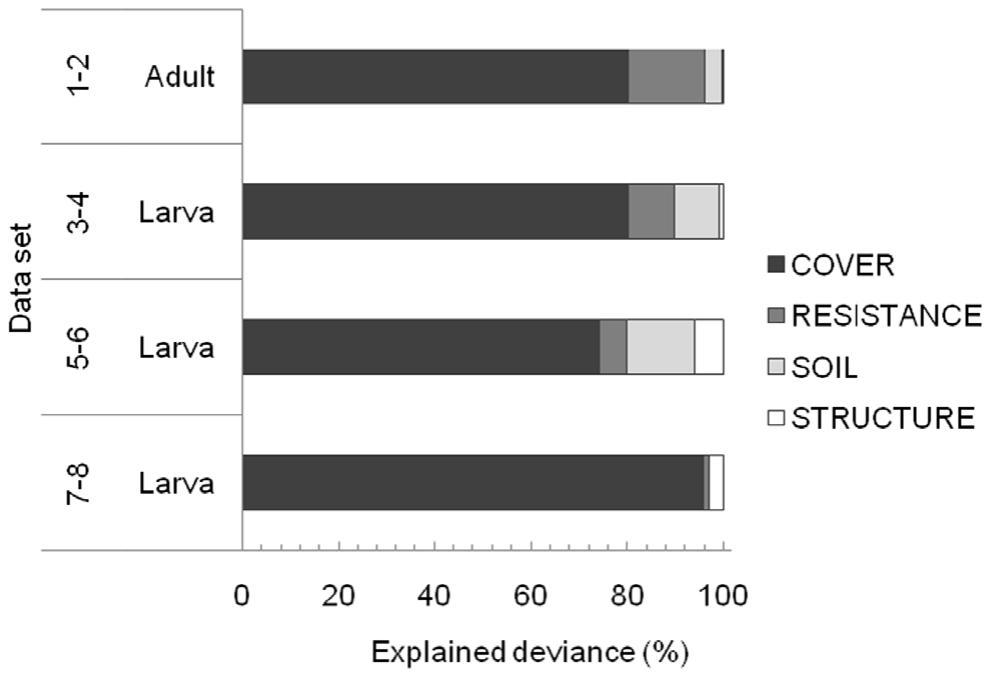
Joint percentage of the total deviance explained by the predictors in the abiotic (ABIOT) minimal adequate models (MAMs). Mean values (*N* = 4) were computed from adult and larval presence/absence (PA) and abundance (AB) models of the two locations (A and B), and individually for each larval sampling scale. Abiotic predictor categories: COVER = percentage cover, RESISTANCE = vegetation resistance, SOIL = soil features, and STRUCTURE = habitat structure; see Table 2.

The number of abiotic predictors in the adult MAMs varied from four to seven (Table S2), with COVER variables accounting for the highest amount of explained deviance (Figure 3). Both adult PA and AB responded negatively to HERB_1 and were benefited by BARESOIL_1. Coarse-scale (1×1 m and 50×50 cm) larval MAMs included between six and 11 predictors, while fine-scale (25×25 cm) larval MAMs contained between two and six predictors (Table S2). At both spatial resolutions, larvae were mostly influenced by COVER variables (Figure 3). In the majority of the coarse-scale models, larvae responded negatively to either HERB_1 or HERB_50 and VACCINIUM _1 or VACCINIUM_50; whereas at the fine scale, larvae were always positively influenced by BARESOIL_25.

**Figure 4:**
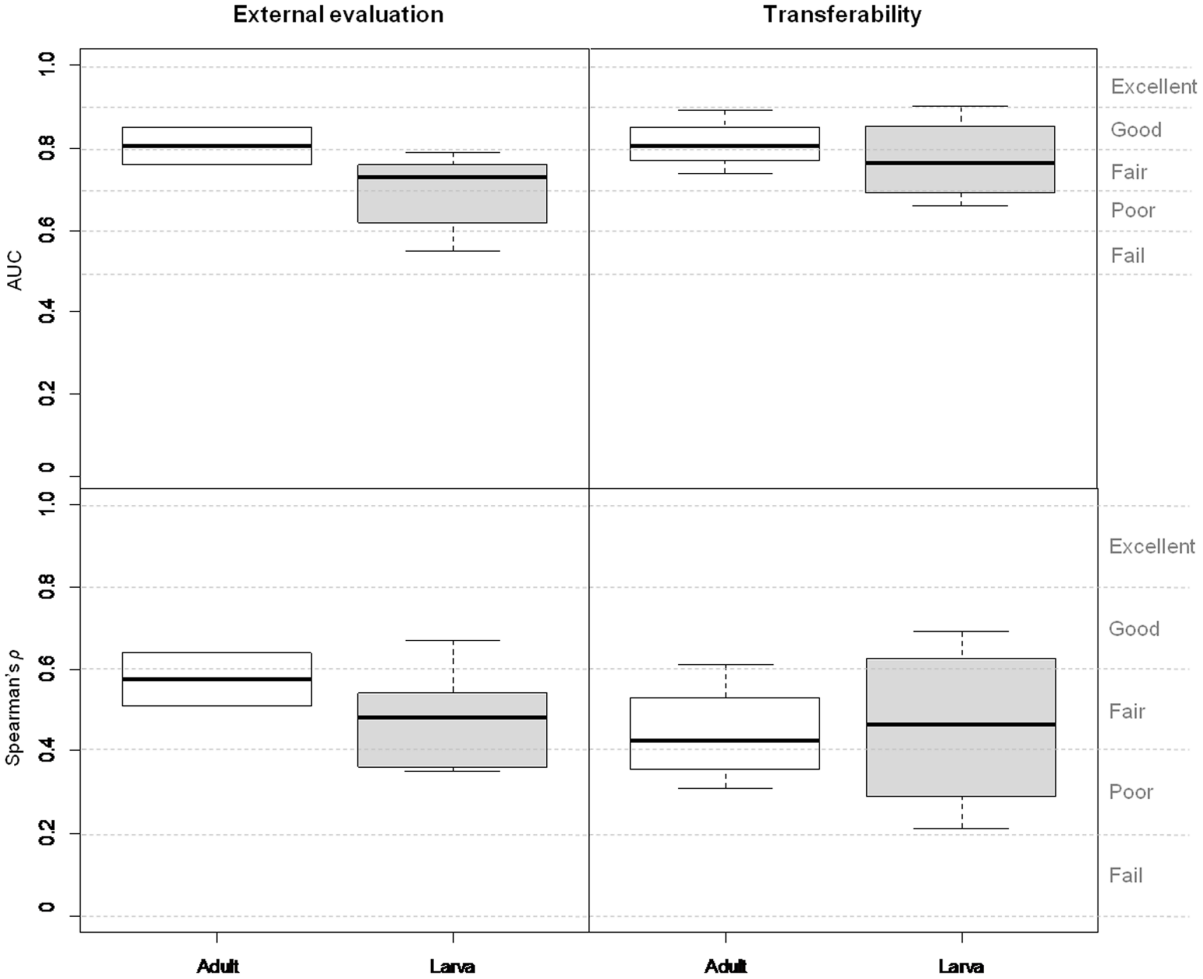
Boxplots of the measures (AUC and Spearman’s *ρ*) of predictive accuracy and transferability. Predictive accuracy measures of the abiotic (ABIOT) minimal adequate models (MAMs) (left) were derived by external evaluation (i.e., two independent calibration-evaluation data sets from locations A and B). Transferability measures of the ABIOT MAMs (right) indicate their cross-applicability between life stages. The plotted values for adult model transferability indicate the ability of adult models to predict larval occurrence. The plotted values for larval model transferability indicate the ability of larval models to predict adult occurrence.

In general, ABIOT MAM predictive accuracy measured by IE was higher than accuracy measured by EE. For adult PA models, IE accuracy values were above ‘good’ threshold values (Table S2), while EE values were above ‘fair’ threshold values (Figure 4). IE accuracy values of the larval PA models were classified as ‘excellent’ at 1×1 m, either ‘good’ or ‘fair’ at 50×50 cm, and ‘fair’ at 25×25 cm. EE accuracy values of larval PA models calibrated in location B were always classified as ‘fair’ at the three spatial scales, while a greater range of values was obtained for models calibrated in location A [i.e., AUC values were classified as ‘fair’ (1×1 m), ‘poor’ (25×25 cm) and ‘fail’ (50×50 cm)]. For adult AB models, IE accuracy values were classified as ‘good’ (Table S2), while EE values were classified as either ‘good’ or ‘fair’ (Figure 4). IE accuracy values of the larval AB models were ‘good’ at 1×1 m, and ‘fair’ at 50×50 and 25×25 cm. EE accuracy values of larval AB models calibrated in location B were either ‘good’ at 1×1 m or ‘fair’ at the finer scales, but ‘fair’ (1×1 m) or ‘poor’ (50×50 and 25×25 cm) in location A.

For both PA and AB data, transferability (T) of the adult ABIOT MAMs was higher than T of the larval ones (Figure 4). When measuring T with the AUC metric, adult models adequately predicted larval occurrences (i.e., AUC values were always above the threshold value of 0.70), while 25% of the larval models failed to predict adult occurrences. But, when measuring T with the *ρ* metric, only 75% of the adult and 50% of the larval models were satisfactorily transferable (i.e., *ρ*>0.40).

Models transferred better from location B to A than *vice versa* (i.e., we found a decrease of 6% for the mean AUC and 24% for the mean *ρ* coefficient when the models were transferred from location A to B). Life stages showed smaller AT than locations. Models transferred slightly better from adult to larva than *vice versa*, as the mean AUC and *ρ* metrics decreased 5% and 2% respectively when models were transferred from larva to adult.

### Biotic Model

The fit of PA models was mostly lower than or equal to that of AB models (Table 3). In 10 of the 16 BIOT MAMs, biotic predictors explained 20–73% of the null deviance. Model fit differed between locations (i.e., greater in A than in B), except for the larval models at 25×25 cm. Comparable mean values of fit were obtained for the adult (0.42±0.26) and larval models at 1×1 m (0.38±0.34) and 50×50 cm (0.51±0.17).

Nearly all adult and larval responses to biotic predictors in the BIOT MAMs were significantly positive (Table S3). The majority of adult MAMs included larva (either PA or AB) of the target species as significant biotic predictor. Six of eight larval MAMs at 1×1 m and 50×50 cm included adult (PA and/or AB) and/or female/male (PA) of the target species as important predictors. Congeneric adult (PA) and larva (PA) were always retained in the MAMs after selection of variables. Similarly, neighbouring larva (AB) was the main, if not the only, biotic predictor in the larval MAMs at the finest scales (50×50 and 25×25 cm).

For six out of eight adult and larval PA models, and for four out of eight AB models, IE accuracy values were above ‘fair’ threshold values (Table S3).

### Contribution to the Full Model

**Figure 5:**
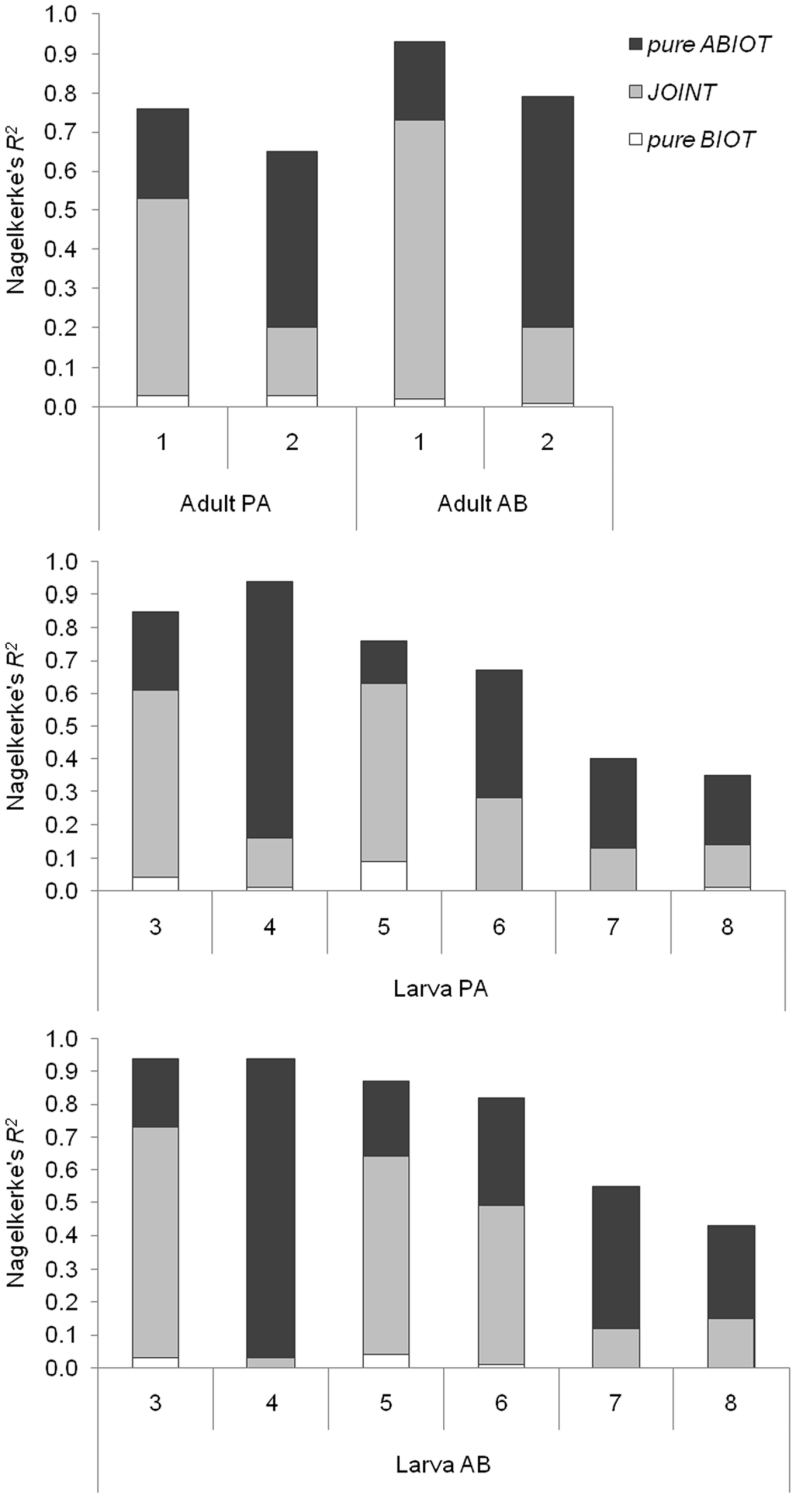
Partition of the goodness of fit measure (Nagelkerke’s *R^2^*) for the modelled data sets. *Pure ABIOT* = independent contribution of the abiotic set of predictors to the total explanatory power, *pure BIOT* = independent contribution of the biotic set, *JOINT* = joint contribution of both predictor sets. PA = presence/absence, AB = abundance.

The inclusion of biotic predictors did not improve model fit substantially (Table 3), and the majority of AIC values of the FULL models were greater than, or very similar to, the values of the ABIOT models (Table 4). The contribution of biotic predictors alone to the model’s explanatory power was negligible (0.02±0.02; Figure 5). Overall, the contribution of abiotic predictors alone (0.37±0.22) was equivalent to that of abiotic and biotic predictors jointly (0.34±0.24). However, the joint contribution (0.48±0.23) was larger than the abiotic contribution (0.24±0.09) in location A, whereas the abiotic contribution (0.49±0.25) was larger than the joint contribution (0.20±0.13) in location B.

**Table 4:** Akaike’s information criterion (AIC) values of the null, abiotic (ABIOT), biotic (BIOT) and combined (FULL) models for each adult and larval presence/absence (PA) and abundance (AB) data set of the target species *Cicindela sylvatica.*

Data set	PA				AB			
	Null	ABIOT	BIOT	FULL	Null	ABIOT	BIOT	FULL
**Adult**								
1	158.59	81.34	104.65	**79.53**	405.32	206.95	282.43	**205.62**
2	154.76	91.27	140.30	**90.24**	270.99	**194.16**	259.81	195.25
**Larva**								
3	166.22	68.12	101.75	**67.05**	477.52	369.77	414.48	**363.40**
4	168.06	**46.89**	158.72	48.88	391.62	**266.17**	391.21	267.81
5	151.84	90.68	86.46	**79.74**	293.42	229.28	247.84	**225.24**
6	122.10	**77.88**	101.04	79.87	217.90	**124.71**	166.76	126.31
7	221.78	**177.48**	NA	179.31	465.24	**405.05**	455.53	406.37
8	204.22	**165.31**	188.19	166.83	296.44	**253.02**	284.56	254.97

Bold face indicates the model with the highest fit (i.e., lowest AIC value).

## Discussion

Numerous processes acting at several spatial scales determine the niche and distributional area of a species [13], [18], particularly for species exhibiting diverse life strategies and needs across life-cycle stages (i.e., ontogenetic niche shift [30]; see [3], [35]). Such complexity is often overlooked when applying niche modelling to conservation (e.g., when identifying priority areas or forecasting responses to climate change), as the majority of models focus on the occurrences of a single life stage (commonly the adult), and are based on variables measured at broad scales and low resolutions (e.g., [64]; but see [4], [65]). For locally dispersing or small-sized species such as the heath tiger beetle, broad-scale (e.g., hundreds of kilometres) models based solely on coarse-grained variables (e.g., climate) may be insufficient to predict true responses to environment alteration at a scale that is relevant to conservation planning [13], [15], [16]. The results of the local scale niche models for the life stages of the heath tiger beetle confirm that: 1) fine-grained abiotic (i.e., vegetation structure) and biotic (i.e., intraspecific interactions) factors are meaningful variables explaining the distribution of co-occurring adults and larvae; and 2) at fine spatial resolution, the effect of biotic interactions on life-stage occurrence is largely correlated with that of the abiotic factors. Despite the different dispersal rates of adults and larvae, both life stages coexist within single locations and exhibit a high degree of spatial aggregation, which explains the significant correlation of adult and larval occurrences in the models. The fact that the biotic niche component is mostly contained in the abiotic signature at the scale of hundreds or tens of meters supports the need for increasing resolution to obtain a more accurate definition of the potential biotic processes affecting life stages [2], [6], [9]. Interaction processes (positive or negative) involving adults may arise at a scale of only few meters [i.e., point (ten to one meters [9]) and observation or sampling unit (less than one meter) scales], and at a scale of centimetres in the case of the sedentary larvae (i.e., vicinity or immediacy scale) (see [40], [41]). For both life stages, the magnitude of the effect of biotic interactions varied between geographical locations, suggesting the spatial structure of the biotic niche component is highly dynamic [12] and likely influenced by density-dependent processes (i.e., the greatest biotic effects corresponded to the location with higher number of presences; see [41]).

Larval and adult stages of the model species inhabit *Calluna vulgaris* heathlands characterised by specific combinations of densely vegetated and bare ground areas. At larger modelling scales (1×1 m and 50×50 cm), the respective extents of these two elements in the suitable habitat constitute the main abiotic factor shaping life-stage occurrences. Bare, open ground devoid of vegetation provides essential sun-exposed surfaces where adults and larvae attain the high body temperature indispensable for prompt motion (i.e., adult running and flying, and larval rising) and for prey detection [39], whereas vegetation patches provide adults with shelter and protection from adverse weather and natural enemies (see [40]). Relatively high transferability of the abiotic niche models between the two life stages confirms that, within the scale of hundreds or tens of meters, the maintenance of such heathland structure is the first requisite for the species’ survival. Assuming the main biotic restrictions of the adult niche have been accounted for in the models (see [66]), accomplishment of this requisite may satisfy both the abiotic and biotic requirements of the adult stage, as long as accessibility to the suitable habitat is ensured.

Several studies on the environmental requirements of tiger beetles have demonstrated that larvae exhibit narrower tolerances than adults do [67]–[69], corroborating observations that adults are scattered fairly evenly over the suitable habitat, while larvae concentrate in a limited number of favourable places (see [40]). The more restricted spatial distribution of the larvae within the suitable habitat may reflect additional environmental needs or further constraints related to larval natural history. Larval spatial distribution is very likely determined by the extremely specific choice of oviposition sites made by the female [70], [71], as larvae seldom move from the sites where they hatched [39], [40], [69]. The smallest scale (25×25 cm) models of the heath tiger beetle evidenced a decrease in the importance of the measured abiotic factors in determining larval occurrences (i.e., lower model fit and predictive accuracy), suggesting the existence of important additional variables acting within a few centimetres (e.g., micro-climate, crowding effects; see [16], [41], [72]). Such a small scale may require the development of a more accurate individual-based approach for assessing the relation between the sedentary larvae and their immediate environment [41]. Yet, sustained significant correlations between neighbouring larvae in the models provide an indication of the potential impact of neighbourhood crowding on the larval niche within a few centimetres. Clearly, this evidence is insufficient to attain full understanding of the complex mechanisms underlying larval interactions (and thus niche restrictions) that involve a variety of adaptive life-history strategies (e.g., prolongation of the developmental period, resistance to starvation, food resource partitioning) [40], [41], [69]. However, it underlines the necessity for a more critical evaluation of the consequences of ignoring niche variation between life stages when making predictions to address species conservation [3], [4].

**Figure 6:**
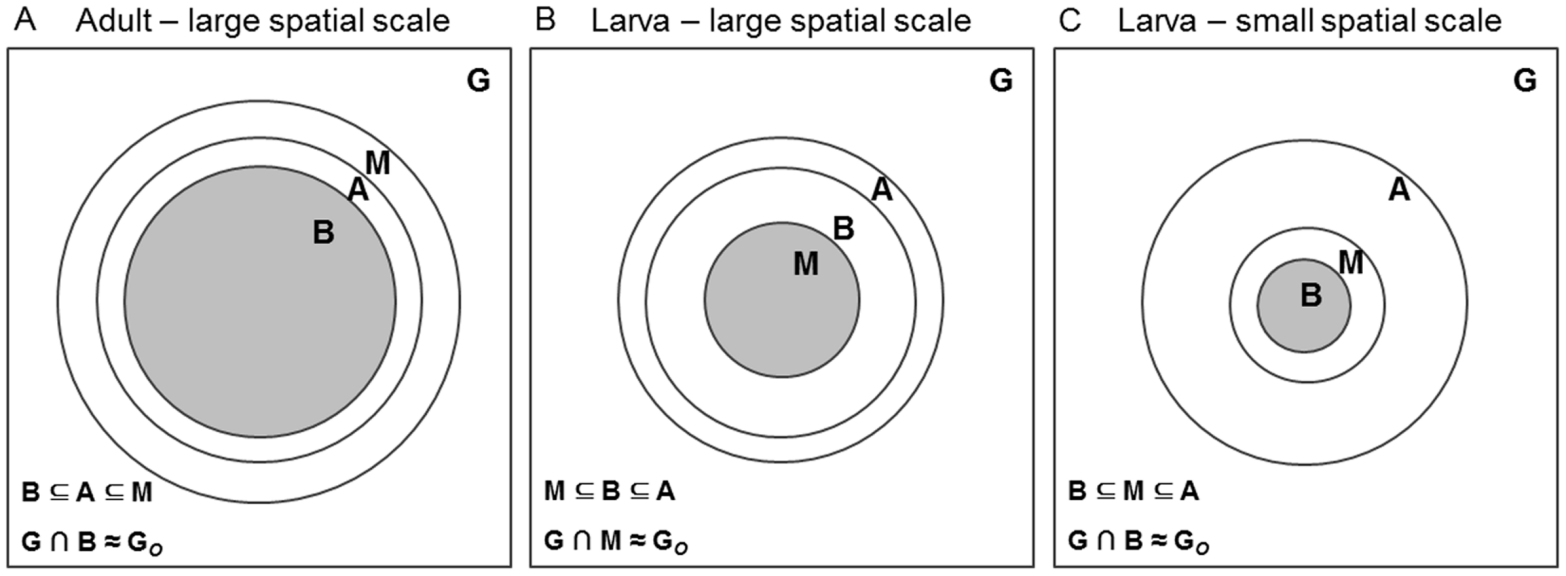
Life-stage modelling results interpreted by a simplified configuration of the BAM (biotic, abiotic, movement) diagram [2], [6], [56]. The regions in the BAM diagram represent areas in the geographic space: G = entire sampling area, A = area with suitable abiotic conditions, B = area with favourable biotic conditions, M = accessible area limited by movement restrictions and dispersal factors. The intersection of B, A and M represents the actual area of occupancy (G*_O_* = shaded area), equivalent to the occupied niche [1], [6]. Panels A and B illustrate the results of the adult and larval models at the scale of hundreds of meters (large spatial scale), respectively. Panel C incorporates the assumption supported by previous works [40], [41] that fundamental biotic constraints involving neighbouring larvae are likely to emerge at the scale of centimetres (small spatial scale). See text for further explanation.

We exemplify three configurations of the **BAM** (biotic, abiotic, movement) diagram (Figure 6; see [1]) to illustrate how integrating life stages and varying resolution may lead to different estimates of areas of occupancy and niches for the heath tiger beetle, at the spatial scale relevant for practical conservation (e.g., disturbance mimicry [73]; reintroduction [68], [74]). The regions in the **BAM** diagram represent areas in the geographic space (**G**) where biotic (**B**) and abiotic (**A**) conditions are suitable for occurrence, and that have been accessible to dispersal or colonisation over time (**M**) [1], [6]. The intersection **B∩A∩M** represents the occupied area of distribution (**G** ***_O_***), equivalent to the occupied niche [1], [6]. The first configuration of the **BAM** diagram (Figure 6A) illustrates the results of the adult models at the scale of hundreds of meters: the areas with suitable abiotic and biotic conditions are nearly coincident (i.e., **A** and **B** regions in the diagram overlap almost completely), and because no substantial restrictions to adult dispersal exist (i.e., the **M** region is large with respect to **A** and **B**), the principal constraint to adult occurrence is lack of favourable environments (the Hutchinson’s Dream scenario as it was called by Saupe *et al.* [75]). The second configuration of the **BAM** diagram (Figure 6B) represents the results of the larval models at the scale of hundreds of meters: the areas with suitable abiotic and biotic conditions match those of the adult (i.e., the **A** and **B** regions of the larval and adult diagrams are identical), but important restrictions to larval dispersal exist (i.e., the **M** region of the larva is reduced), such as the availability of proper oviposition sites and the limited larval motion, implying that not all suitable areas for the larvae are occupied (the Wallace’s Dream scenario [75]). The third configuration of the **BAM** diagram (Figure 6C) incorporates the assumption supported by previous works [40], [41] that fundamental biotic constraints involving neighbouring larvae are likely to emerge at the scale of centimetres (i.e., the **B** region is substantially reduced), which may limit larval occurrence within the accessible region **M**. The key mechanisms of larval spatial aggregation (e.g., avoidance of natural enemies, food patchiness, competition processes) have yet to be understood, but for either positive (beneficial) or negative (detrimental) interactions a threshold number or density of neighbouring larvae may be needed for population survival [41], conditioning the implementation of management actions (e.g., the number and placement of translocated larvae [74]). Tiger beetles spend the majority of their life cycle in the larval stage [39], and thus management is necessary for both adults and larvae to effectively enhance viable populations of threatened species. This stresses the importance of integrating early life stages when modelling niches so as to reach accurate interpretations of the factors underlying species distributions [4] and to adequately translate these into conservation planning (e.g., [74]).

## Supporting Information

Table S1 **Number of sampling points and number of captured adults and monitored larvae of the two tiger beetle species per habitat type in each location.** Target species = *Cicindela sylvatica*, congeneric species = *Cicindela campestris*. Adult captures correspond to 63 and 49 marked individuals in locations A and B, respectively. Values in parentheses represent the number of sampling points in which adults and larvae were present. BARE_CUSHION = *Calluna vulgaris* (L.) Hull heathland characterised by the combination of dense vegetation arranged in a cushion-like structure and large patches of bare ground, BARE_SPOT = *C. vulgaris* heathland dominated by dense vegetation interrupted by small spots of bare ground, BARE_PATH = *C. vulgaris* heathland consisting of dense vegetation interrupted by bare ground paths caused by cattle trampling, BURNED = *C. vulgaris* heathland burned in September 2010 to create pastureland and improve forage quality, DENSE = *C. vulgaris* heathland consisting of dense continuous vegetation, GRASSLAND = upland siliceous grassland used as summer pasture, SHRUBLAND = shrub formation consisting of *Cytisus purgans* (L.) Boiss, *Cytisus scoparius* (L.) Link and *Genista florida* L.
(DOCX)

Table S2 **Abiotic (ABIOT) minimal adequate model (MAM) results for each adult and larval presence/absence (PA) and abundance (AB) data set.** Final GLM coefficient estimates (log values) and standard errors (SEs) are indicated. Residual deviance is the amount of variation not explained by the predictors. *P* values smaller than 0.05 (χ^2^ distribution) are in bold face. Measures of model predictive accuracy (AUC and Spearman’s *ρ*) were derived by internal evaluation (i.e., 2-fold cross-validations of a single data set). For predictor abbreviations see Table 2.
(DOCX)

Table S3 **Biotic (BIOT) minimal adequate model (MAM) results for each adult and larval presence/absence (PA) and abundance (AB) data set.** Final GLM coefficient estimates (log values) and standard errors (SEs) are indicated. Residual deviance is the amount of variation not explained by the predictors. *P* values smaller than 0.05 (χ^2^ distribution) are in bold face. Measures of model predictive accuracy (AUC and Spearman’s *ρ*) were derived by internal evaluation (i.e., 2-fold cross-validations of a single data set). Target species = *Cicindela sylvatica*, congeneric species = *Cicindela campestris*. Larva_ 2 = second larval instar.
(DOCX)
