# Supplementary material for: Integrating Life Stages into Ecological Niche Models: A Case Study on Tiger Beetles
Source: PLoS One. 2013 Jul 23;8(7):e70038. doi: 10.1371/journal.pone.0070038 (PMC3720956; doi:10.1371/journal.pone.0070038)
Supplement: Table S1 — Number of sampling points and number of captured adults and monitored larvae of the two tiger beetle species per habitat type in each location. Target species = Cicindela sylvatica, congeneric species = Cicindela campestris. Adult captures correspond to 63 and 49 marked individuals in locations A and B, respectively. Values in parentheses represent the number of sampling points in which adults and larvae were present. BARE_CUSHION = Calluna vulgaris (L.) Hull heathland characterised by the combination of dense vegetation arranged in a cushion-like structure and large patches of bare ground, BARE_SPOT = C. vulgaris heathland dominated by dense vegetation interrupted by small spots of bare ground, BARE_PATH = C. vulgaris heathland consisting of dense vegetation interrupted by bare ground paths caused by cattle trampling, BURNED = C. vulgaris heathland burned in September 2010 to create pastureland and improve forage quality, DENSE = C. vulgaris heathland consisting of dense continuous vegetation, GRASSLAND = upland siliceous grassland used as summer pasture, SHRUBLAND = shrub formation consisting of Cytisus purgans (L.) Boiss, Cytisus scoparius (L.) Link and Genista florida L. (DOCX) [file pone.0070038.s001.docx]

**Table S1.** Number of sampling points and number of captured adults and monitored larvae of the two tiger beetle species per habitat type in each location.

|  |  | **Target species** | | **Congeneric species** | |
| --- | --- | --- | --- | --- | --- |
| **Habitat type** | **Sampling points** | **Adult** | **Larva** | **Adult** | **Larva** |
| **Location A** |  |  |  |  |  |
| BARE_CUSHION | 60 | 115 (40) | 378 (50) | 41 (28) | 35 (24) |
| BARE_SPOT | 15 | 3 (3) | 5 (2) | ‐ | 2 (1) |
| DENSE | 15 | ‐ | ‐ | ‐ | ‐ |
| GRASSLAND | 15 | ‐ | ‐ | ‐ | ‐ |
| SHRUBLAND | 15 | ‐ | ‐ | ‐ | ‐ |
| **Location B** |  |  |  |  |  |
| BARE_PATH | 60 | 48 (29) | 174 (57) | 22 (16) | ‐ |
| BURNED | 15 | 34 (11) | ‐ | 8 (6) | ‐ |
| DENSE | 15 | ‐ | ‐ | ‐ | ‐ |
| GRASSLAND | 15 | ‐ | ‐ | ‐ | ‐ |
| SHRUBLAND | 15 | ‐ | ‐ | ‐ | ‐ |

Target species=*Cicindela sylvatica*, congeneric species=*Cicindela campestris*. Adult captures correspond to 63 and 49 marked individuals in locations A and B, respectively. Values in parentheses represent the number of sampling points in which adults and larvae were present. BARE_CUSHION=*Calluna vulgaris* (L.) Hull heathland characterised by the combination of dense vegetation arranged in a cushion-like structure and large patches of bare ground, BARE_SPOT=*C. vulgaris* heathland dominated by dense vegetation interrupted by small spots of bare ground, BARE_PATH=*C. vulgaris* heathland consisting of dense vegetation interrupted by bare ground paths caused by cattle trampling, BURNED=*C. vulgaris* heathland burned in September 2010 to create pastureland and improve forage quality, DENSE=*C. vulgaris* heathland consisting of dense continuous vegetation, GRASSLAND=upland siliceous grassland used as summer pasture, SHRUBLAND=shrub formation consisting of *Cytisus purgans* (L.) Boiss, *Cytisus scoparius* (L.) Link and *Genista florida* L.
