# Supplementary material for: Integrating Life Stages into Ecological Niche Models: A Case Study on Tiger Beetles
Source: PLoS One. 2013 Jul 23;8(7):e70038. doi: 10.1371/journal.pone.0070038 (PMC3720956; doi:10.1371/journal.pone.0070038)
Supplement: Table S2 — Abiotic (ABIOT) minimal adequate model (MAM) results for each adult and larval presence/absence (PA) and abundance (AB) data set. Final GLM coefficient estimates (log values) and standard errors (SEs) are indicated. Residual deviance is the amount of variation not explained by the predictors. P values smaller than 0.05 (χ2 distribution) are in bold face. Measures of model predictive accuracy (AUC and Spearman’s ρ) were derived by internal evaluation (i.e., 2-fold cross-validations of a single data set). For predictor abbreviations see Table 2. (DOCX) [file pone.0070038.s002.docx]

**Table S2.** Abiotic (ABIOT) minimal adequate model (MAM) results for each adult and larval presence/absence (PA) and abundance (AB) data set.

| **Data set** | **PA** |  |  |  |  |  |  | **AB** |  |  |  |  | |  |  |
| --- | --- | --- | --- | --- | --- | --- | --- | --- | --- | --- | --- | --- | --- | --- | --- |
|  |  |  |  |  | **Coefficients** | |  |  |  |  |  | **Coefficients** | | |  |
|  | **Null deviance** | **Predictors** | **Residual deviance** | ***P* value** | **Estimate** | **SE** | **AUC** | **Null deviance** | **Predictors** | **Residual deviance** | ***P* value** | **Estimate** | **SE** | | **Spearman's *ρ*** |
| **Adult** |  |  |  |  |  |  |  |  |  |  |  |  |  | |  |
| 1 | 156.59 | (Intercept) |  |  | 21.27 | 11.20 | 0.91 | 285.87 | (Intercept) |  |  | 3.24 | 0.50 | | 0.73 |
|  |  | BARESOIL_1 | 85.93 | **<0.001** | 0.05 | 0.03 |  |  | VACCINIUM_50 | 245.85 | **<0.001** | -0.17 | 0.06 | |  |
|  |  | VACCINIUM_50 | 84.51 | 0.233 | -0.20 | 0.14 |  |  | MOSS_50 | 235.00 | **<0.001** | -0.04 | 0.03 | |  |
|  |  | MOSS_50 | 83.25 | 0.262 | -0.09 | 0.05 |  |  | HERB_1 | 109.88 | **<0.001** | -0.07 | 0.02 | |  |
|  |  | HERB_1 | 72.27 | **<0.001** | -0.06 | 0.04 |  |  | LITTER_1 | 86.74 | **<0.001** | -0.03 | 0.01 | |  |
|  |  | LITTER_1 | 70.61 | 0.198 | -0.04 | 0.03 |  |  | SQUARE_N | 83.43 | 0.069 | -0.06 | 0.03 | |  |
|  |  | DIST_OPEN | 69.04 | 0.211 | -0.01 | 0.01 |  |  | SOIL_OM | 75.56 | **0.005** | -0.10 | 0.04 | |  |
|  |  | SOIL_PH | 65.34 | 0.054 | -4.48 | 2.44 |  |  | SOIL_0.063 | 73.49 | 0.151 | -0.05 | 0.04 | |  |
| 2 | 152.76 | (Intercept) |  |  | 2.47 | 0.66 | 0.88 | 192.00 | (Intercept) |  |  | -0.79 | 0.56 | | 0.65 |
|  |  | HERB_1 | 117.76 | **<0.001** | -0.06 | 0.02 |  |  | BARESOIL_1 | 101.87 | **<0.001** | 0.02 | 0.01 | |  |
|  |  | SQUARE_S | 94.77 | **<0.001** | -0.16 | 0.06 |  |  | HERB_1 | 92.64 | **0.002** | -0.03 | 0.01 | |  |
|  |  | SQUARE_N | 84.73 | **0.002** | -0.40 | 0.18 |  |  | SQUARE_S | 82.03 | **0.001** | -0.09 | 0.04 | |  |
|  |  | LITTER_DEPTH | 81.27 | 0.063 | -3.45 | 2.23 |  |  | SQUARE_N | 74.07 | **0.005** | -0.24 | 0.11 | |  |
| **Larva** |  |  |  |  |  |  |  |  |  |  |  |  |  | |  |
| 3 | 164.22 | (Intercept) |  |  | 9.81 | 4.32 | 0.93 | 295.26 | (Intercept) |  |  | 5.91 | 0.77 | | 0.76 |
|  |  | VACCINIUM_1 | 160.13 | **0.043** | -0.10 | 0.06 |  |  | HERB_1 | 147.01 | **<0.001** | -0.11 | 0.03 | |  |
|  |  | HERB_1 | 95.20 | **<0.001** | -0.13 | 0.04 |  |  | LITTER_1 | 102.81 | **<0.001** | -0.05 | 0.01 | |  |
|  |  | LICHEN_1 | 87.81 | **0.007** | 0.39 | 0.14 |  |  | WOOD_1 | 102.43 | 0.540 | 0.10 | 0.06 | |  |
|  |  | LITTER_1 | 60.21 | **<0.001** | -0.14 | 0.04 |  |  | DIST_OPEN | 99.55 | 0.089 | -0.00 | 0.00 | |  |
|  |  | WOOD_1 | 56.54 | 0.055 | 0.22 | 0.11 |  |  | SOIL_OM | 82.70 | **<0.001** | -0.19 | 0.04 | |  |
|  |  | SOIL_0.50 | 56.09 | 0.502 | -0.26 | 0.18 |  |  | SOIL_0.063 | 80.00 | 0.100 | -0.09 | 0.05 | |  |
|  |  | SOIL_0.063 | 52.12 | **0.047** | -0.34 | 0.18 |  |  |  |  |  |  |  | |  |
| 4 | 166.06 | (Intercept) |  |  | 19.66 | 7.93 | 0.92 | 326.32 | (Intercept) |  |  | 4.55 | 1.00 | | 0.80 |
|  |  | VACCINIUM_1 | 161.15 | **0.027** | -0.26 | 0.09 |  |  | VACCINIUM_1 | 309.01 | **<0.001** | -0.04 | 0.02 | |  |
|  |  | HERB_1 | 110.15 | **<0.001** | -0.05 | 0.03 |  |  | HERB_1 | 182.99 | **<0.001** | -0.03 | 0.01 | |  |
|  |  | LICHEN_1 | 109.66 | 0.482 | 0.25 | 0.13 |  |  | LICHEN_1 | 182.98 | 0.912 | 0.02 | 0.01 | |  |
|  |  | LITTER_1 | 101.50 | **0.004** | -0.15 | 0.06 |  |  | LITTER_1 | 173.91 | **0.003** | -0.03 | 0.01 | |  |
|  |  | ROOT_1 | 94.11 | **0.007** | 1.03 | 0.58 |  |  | WOOD_1 | 131.10 | **<0.001** | -0.03 | 0.02 | |  |
|  |  | STONE_1 | 91.38 | 0.099 | -0.13 | 0.07 |  |  | SEDUM_1 | 118.13 | **<0.001** | 0.07 | 0.04 | |  |
|  |  | HEIGHT | 65.70 | **<0.001** | 0.50 | 0.16 |  |  | HEIGHT | 111.84 | **0.012** | 0.05 | 0.02 | |  |
|  |  | SQUARE_E | 57.20 | **0.004** | -0.21 | 0.12 |  |  | DIST_OPEN | 105.87 | **0.015** | -0.00 | 0.00 | |  |
|  |  | SQUARE_N | 39.33 | **<0.001** | -0.41 | 0.17 |  |  | LITTER_DEPTH | 86.31 | **<0.001** | -1.77 | 0.74 | |  |
|  |  | SOIL_1 | 26.80 | **<0.001** | -0.35 | 0.19 |  |  | SOIL_HUMID | 81.14 | **0.023** | -0.02 | 0.01 | |  |
|  |  | SOIL_0.50 | 22.89 | **0.048** | -0.76 | 0.40 |  |  | SOIL_0.50 | 74.30 | **0.009** | -0.12 | 0.05 | |  |
| 5 | 149.84 | (Intercept) |  |  | 42.18 | 12.92 | 0.87 | 211.28 | (Intercept) |  |  | 13.72 | 5.41 | | 0.59 |
|  |  | HERB_50 | 120.59 | **<0.001** | -0.07 | 0.02 |  |  | VACCINIUM_50 | 195.21 | **<0.001** | -0.10 | 0.05 | |  |
|  |  | LITTER_1 | 98.35 | **<0.001** | -0.08 | 0.03 |  |  | HERB_50 | 138.08 | **<0.001** | -0.03 | 0.02 | |  |
|  |  | HEIGHT | 94.78 | 0.059 | -0.10 | 0.05 |  |  | LITTER_1 | 122.99 | **<0.001** | -0.06 | 0.02 | |  |
|  |  | SOIL_HUMID | 94.67 | 0.740 | 0.06 | 0.04 |  |  | ROOT_1 | 117.17 | **0.016** | 0.07 | 0.02 | |  |
|  |  | SOIL_PH | 87.88 | **0.009** | -6.47 | 2.32 |  |  | COVER_W | 103.01 | **<0.001** | 0.02 | 0.01 | |  |
|  |  | SOIL_OM | 76.70 | **<0.001** | -0.39 | 0.14 |  |  | SQUARE_N | 97.30 | **0.017** | 0.06 | 0.03 | |  |
|  |  | SOIL_0.50 | 76.56 | 0.714 | -0.22 | 0.16 |  |  | DIST_OPEN | 93.19 | **0.042** | -0.00 | 0.00 | |  |
|  |  | SOIL_0.063 | 72.68 | **0.049** | -0.30 | 0.16 |  |  | SOIL_PH | 85.57 | **0.006** | -2.94 | 1.11 | |  |
|  |  |  |  |  |  |  |  |  | SOIL_OM | 77.70 | **0.005** | -0.19 | 0.06 | |  |
|  |  |  |  |  |  |  |  |  | SOIL_0.50 | 70.84 | **0.009** | 0.15 | 0.06 | |  |
| 6 | 120.10 | (Intercept) |  |  | 8.70 | 4.52 | 0.79 | 158.62 | (Intercept) |  |  | 7.38 | 1.99 | | 0.51 |
|  |  | SHRUB_50 | 110.76 | **0.002** | -0.07 | 0.03 |  |  | SHRUB_50 | 143.88 | **<0.001** | -0.05 | 0.01 | |  |
|  |  | VACCINIUM_50 | 107.53 | 0.072 | -0.38 | 0.21 |  |  | VACCINIUM_50 | 134.80 | **0.003** | -0.13 | 0.10 | |  |
|  |  | LICHEN_50 | 104.16 | 0.066 | 0.06 | 0.05 |  |  | LICHEN_50 | 125.08 | **0.002** | 0.05 | 0.01 | |  |
|  |  | LITTER_50 | 102.96 | 0.274 | -0.05 | 0.04 |  |  | ROOT_50 | 104.94 | **<0.001** | 0.37 | 0.13 | |  |
|  |  | ROOT_50 | 88.33 | **<0.001** | 0.86 | 0.39 |  |  | WOOD_1 | 88.81 | **<0.001** | -0.07 | 0.04 | |  |
|  |  | STONE_50 | 87.37 | 0.328 | -0.06 | 0.03 |  |  | SEDUM_1 | 77.64 | **<0.001** | 0.09 | 0.06 | |  |
|  |  | HERB_1 | 80.51 | **0.009** | -0.03 | 0.02 |  |  | DIST_OPEN | 59.24 | **<0.001** | -0.00 | 0.00 | |  |
|  |  | WOOD_1 | 61.37 | **<0.001** | -0.21 | 0.09 |  |  | SOIL_0.50 | 49.43 | **0.002** | -0.32 | 0.10 | |  |
|  |  | HEIGHT | 58.91 | 0.117 | 0.11 | 0.08 |  |  |  |  |  |  |  | |  |
|  |  | DIST_OPEN | 56.27 | 0.104 | -0.00 | 0.00 |  |  |  |  |  |  |  | |  |
|  |  | SOIL_0.50 | 53.88 | 0.123 | -0.34 | 0.23 |  |  |  |  |  |  |  | |  |
| 7 | 219.78 | (Intercept) |  |  | -4.83 | 1.16 | 0.78 | 215.76 | (Intercept) |  |  | -4.09 | 0.69 | | 0.53 |
|  |  | BARESOIL_25 | 177.55 | **<0.001** | 0.03 | 0.01 |  |  | BARESOIL_25 | 148.55 | **<0.001** | 0.04 | 0.01 | |  |
|  |  | WOOD_25 | 173.46 | **0.043** | 0.12 | 0.05 |  |  | CALLUNA_1 | 146.61 | 0.163 | 0.02 | 0.01 | |  |
|  |  | VACCINIUM_1 | 169.09 | **0.037** | 0.21 | 0.12 |  |  | VACCINIUM_1 | 132.65 | **<0.001** | 0.23 | 0.06 | |  |
|  |  | MOSS_1 | 169.09 | 0.989 | 0.13 | 0.08 |  |  |  |  |  |  |  | |  |
|  |  | SQUARE_E | 166.61 | 0.115 | -0.20 | 0.10 |  |  |  |  |  |  |  | |  |
|  |  | DIST_OPEN | 163.47 | 0.077 | 0.01 | 0.00 |  |  |  |  |  |  |  | |  |
| 8 | 202.22 | (Intercept) |  |  | -4.57 | 0.85 | 0.79 | 186.92 | (Intercept) |  |  | -3.87 | 0.63 | | 0.47 |
|  |  | BARESOIL_25 | 167.16 | **<0.001** | 0.04 | 0.01 |  |  | BARESOIL_25 | 138.99 | **<0.001** | 0.04 | 0.01 | |  |
|  |  | ROOT_25 | 163.52 | 0.057 | 0.14 | 0.08 |  |  | SEDUM_25 | 128.24 | **0.001** | 0.06 | 0.02 | |  |
|  |  | SEDUM_25 | 158.36 | **0.023** | 0.09 | 0.04 |  |  |  |  |  |  |  | |  |
|  |  | OPEN_3 | 155.31 | 0.081 | 0.29 | 0.16 |  |  |  |  |  |  |  | |  |

Final GLM coefficient estimates (log values) and standard errors (SEs) are indicated. Residual deviance is the amount of variation not explained by the predictors. *P* values smaller than 0.05 (χ^2^ distribution) are in bold face. Measures of model predictive accuracy (AUC and Spearman’s *ρ*) were derived by internal evaluation (i.e., 2-fold cross-validations of a single data set). For predictor abbreviations see Table 2.
