# Supplementary material for: Integrating Life Stages into Ecological Niche Models: A Case Study on Tiger Beetles
Source: PLoS One. 2013 Jul 23;8(7):e70038. doi: 10.1371/journal.pone.0070038 (PMC3720956; doi:10.1371/journal.pone.0070038)
Supplement: Table S3 — Biotic (BIOT) minimal adequate model (MAM) results for each adult and larval presence/absence (PA) and abundance (AB) data set. Final GLM coefficient estimates (log values) and standard errors (SEs) are indicated. Residual deviance is the amount of variation not explained by the predictors. P values smaller than 0.05 (χ2 distribution) are in bold face. Measures of model predictive accuracy (AUC and Spearman’s ρ) were derived by internal evaluation (i.e., 2-fold cross-validations of a single data set). Target species = Cicindela sylvatica, congeneric species = Cicindela campestris. Larva_ 2 = second larval instar. (DOCX) [file pone.0070038.s003.docx]

**Table S3.** Biotic (BIOT) minimal adequate model (MAM) results for each adult and larval presence/absence (PA) and abundance (AB) data set.

| **Data set** | **PA** |  |  |  |  | |  |  | **AB** |  |  |  |  | |  |  |
| --- | --- | --- | --- | --- | --- | --- | --- | --- | --- | --- | --- | --- | --- | --- | --- | --- |
|  |  |  |  |  | **Coefficients** | | |  |  |  |  |  | **Coefficients** | | |  |
|  | **Null deviance** | **Predictors** | **Residual deviance** | ***P* value** | **Estimate** | **SE** | | **AUC** | **Null deviance** | **Predictors** | **Residual deviance** | ***P* value** | **Estimate** | **SE** | | **Spearman's *ρ*** |
| **Adult** |  |  |  |  |  |  | |  |  |  |  |  |  |  | |  |
| 1 | 156.59 | (Intercept) |  |  | -1.92 | 0.33 | | 0.86 | 285.87 | (Intercept) |  |  | -1.50 | 0.25 | | 0.67 |
|  |  | Larva AB | 104.60 | **<0.001** | 0.38 | 0.10 | |  |  | Larva PA | 190.76 | **<0.001** | 1.24 | 0.33 | |  |
|  |  | Congeneric adult PA | 98.65 | **0.015** | 1.51 | 0.62 | |  |  | Larva_2 AB | 183.62 | **0.008** | 0.09 | 0.03 | |  |
|  |  |  |  |  |  |  | |  |  | Congeneric adult PA | 156.98 | **<0.001** | 1.08 | 0.22 | |  |
| 2 | 152.76 | (Intercept) |  |  | -1.50 | 0.33 | | 0.75 | 112.34 | (Intercept) |  |  | -0.80 | 0.19 | | 0.38 |
|  |  | Larva PA | 145.32 | **0.006** | 0.90 | 0.43 | |  |  | Congeneric adult PA | 97.18 | **<0.001** | 1.35 | 0.36 | |  |
|  |  | Congeneric adult PA | 134.30 | **<0.001** | 1.68 | 0.52 | |  |  |  |  |  |  |  | |  |
| **Larva** |  |  |  |  |  |  | |  |  |  |  |  |  |  | |  |
| 3 | 164.22 | (Intercept) |  |  | -1.83 | 0.35 | | 0.88 | 225.56 | (Intercept) |  |  | -0.82 | 0.21 | | 0.79 |
|  |  | Adult PA | 122.61 | **<0.001** | 2.83 | 1.21 | |  |  | Male PA | 134.54 | **<0.001** | 2.49 | 0.28 | |  |
|  |  | Female PA | 120.56 | 0.151 | -3.74 | 1.41 | |  |  | Congeneric larva PA | 109.57 | **<0.001** | 1.47 | 0.31 | |  |
|  |  | Adult AB | 114.42 | **0.013** | 1.08 | 0.46 | |  |  |  |  |  |  |  | |  |
|  |  | Congeneric larva PA | 91.75 | **<0.001** | 3.04 | 0.73 | |  |  |  |  |  |  |  | |  |
| 4 | 166.06 | (Intercept) |  |  | -0.46 | 0.23 | | 0.67 | 114.30 | (Intercept) |  |  | 0.18 | 0.19 | | 0.27 |
|  |  | Adult PA | 158.61 | **0.006** | 2.22 | 0.75 | |  |  | Adult PA | 111.84 | 0.117 | 0.49 | 0.31 | |  |
|  |  | Male PA | 158.61 | 0.973 | 2.07 | 1.18 | |  |  |  |  |  |  |  | |  |
|  |  | Adult AB | 150.72 | **0.005** | -1.08 | 0.47 | |  |  |  |  |  |  |  | |  |
| 5 | 149.84 | (Intercept) |  |  | -2.38 | 0.39 | | 0.92 | 164.24 | (Intercept) |  |  | -1.58 | 0.26 | | 0.75 |
|  |  | Female PA | 130.61 | **<0.001** | -2.62 | 1.37 | |  |  | Male PA | 99.81 | **<0.001** | 1.56 | 0.39 | |  |
|  |  | Adult AB | 100.05 | **<0.001** | 1.21 | 0.40 | |  |  | Neighbouring larva AB | 87.15 | **<0.001** | 0.12 | 0.03 | |  |
|  |  | Neighbouring larva AB | 78.46 | **<0.001** | 0.52 | 0.15 | |  |  |  |  |  |  |  | |  |
| 6 | 120.10 | (Intercept) |  |  | -2.34 | 0.37 | | 0.80 | 158.62 | (Intercept) |  |  | -1.84 | 0.23 | | 0.49 |
|  |  | Neighbouring larva AB | 97.04 | **<0.001** | 0.67 | 0.17 | |  |  | Neighbouring larva AB | 105.48 | **<0.001** | 0.41 | 0.05 | |  |
| 7 | 219.78 | (Intercept) |  |  | -1.05 | 0.27 | | 0.69 | 154.66 | (Intercept) |  |  | -0.53 | 0.21 | | 0.36 |
|  |  | Neighbouring larva AB | 203.13 | **<0.001** | 0.13 | 0.03 | |  |  | Neighbouring larva AB | 142.27 | **<0.001** | 0.09 | 0.02 | |  |
| 8 | 202.22 | (Intercept) |  |  | -1.82 | 0.28 | | 0.72 | 140.32 | (Intercept) |  |  | -1.56 | 0.23 | | 0.34 |
|  |  | Neighbouring larva AB | 184.19 | **<0.001** | 0.31 | 0.08 | |  |  | Neighbouring larva AB | 125.56 | **<0.001** | 0.23 | 0.06 | |  |

Final GLM coefficient estimates (log values) and standard errors (SEs) are indicated. Residual deviance is the amount of variation not explained by the predictors. *P* values smaller than 0.05 (χ^2^ distribution) are in bold face. Measures of model predictive accuracy (AUC and Spearman’s *ρ*) were derived by internal evaluation (i.e., 2-fold cross-validations of a single data set). Target species=*Cicindela sylvatica*, congeneric species=*Cicindela campestris*. Larva_ 2=second larval instar.
